# Poly[octa-μ-aqua-tetra­aqua­bis(μ_4_-5-sulfonatobenzene-1,3-dicarboxyl­ato)nickel(II)tetra­sodium]

**DOI:** 10.1107/S1600536809009489

**Published:** 2009-03-25

**Authors:** Bing-Yu Zhang, Jing-Jing Nie, Duan-Jun Xu

**Affiliations:** a Department of Chemistry, Zhejiang University, People’s Republic of China

## Abstract

In the crystal structure of the title compound, [Na_4_Ni(C_8_H_3_O_7_S)_2_(H_2_O)_12_]_*n*_, the Ni^II^ cation occupies an inversion centre and is coordinated by the carboxyl groups of the sulfoisophthalate trianions and water mol­ecules in a distorted octa­hedral geometry. Two independent Na^I^ atoms are connected by the carboxyl and sulfonate groups of the sulfoisophthalate ligands anions and water mol­ecules in a distorted octa­hedral geometry. The sulfoisophthalate ligands and coordinated water mol­ecules bridge the Ni^II^ and Na^I^ cations, forming a three-dimensional polymeric structure. Weak π–π stacking is present between parallel benzene rings [centroid–centroid distance = 3.9349 (10) Å]. Extensive O—H⋯O and C—H⋯O hydrogen bonding helps to stabilize the crystal structure.

## Related literature

For general background, see: Su & Xu (2004 ▶); Pan *et al.* (2006 ▶). For the isotypic structure of the Co analogue, see: Zhang *et al.* (2009 ▶).
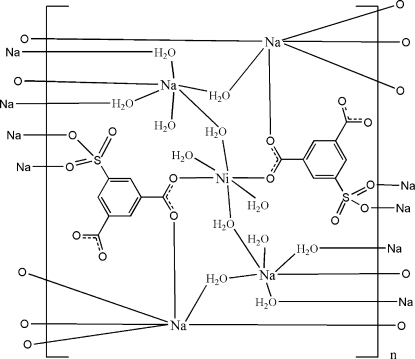


## Experimental

### 

#### Crystal data

[Na_4_Ni(C_8_H_3_O_7_S)_2_(H_2_O)_12_]
*M* *_r_* = 853.19
Monoclinic, 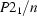

*a* = 7.8770 (9) Å
*b* = 17.229 (2) Å
*c* = 11.7474 (13) Å
β = 93.292 (4)°
*V* = 1591.7 (3) Å^3^
*Z* = 2
Mo *K*α radiation
μ = 0.90 mm^−1^
*T* = 295 K
0.30 × 0.22 × 0.20 mm

#### Data collection

Rigaku R-AXIS RAPID IP diffractometer
Absorption correction: multi-scan (*ABSCOR*; Higashi, 1995 ▶) *T*_min_ = 0.770, *T*_max_ = 0.835
17574 measured reflections
3107 independent reflections
2866 reflections with *I* > 2σ(*I*)
*R*_int_ = 0.023

#### Refinement

*R*[*F* ^2^ > 2σ(*F* ^2^)] = 0.025
*wR*(*F* ^2^) = 0.071
*S* = 1.08
3107 reflections
223 parameters
H-atom parameters constrained
Δρ_max_ = 0.42 e Å^−3^
Δρ_min_ = −0.40 e Å^−3^

### 

Data collection: *PROCESS-AUTO* (Rigaku, 1998 ▶); cell refinement: *PROCESS-AUTO*; data reduction: *CrystalStructure* (Rigaku/MSC, 2002 ▶); program(s) used to solve structure: *SHELXS97* (Sheldrick, 2008 ▶); program(s) used to refine structure: *SHELXL97* (Sheldrick, 2008 ▶); molecular graphics: *ORTEP-3 for Windows* (Farrugia, 1997 ▶); software used to prepare material for publication: *WinGX* (Farrugia, 1999 ▶).

## Supplementary Material

Crystal structure: contains datablocks I, global. DOI: 10.1107/S1600536809009489/ng2561sup1.cif

Structure factors: contains datablocks I. DOI: 10.1107/S1600536809009489/ng2561Isup2.hkl

Additional supplementary materials:  crystallographic information; 3D view; checkCIF report

## Figures and Tables

**Table 1:** Selected bond lengths (Å)

Ni—O1	2.0252 (11)
Ni—O8	2.0731 (14)
Ni—O9	2.0727 (11)

**Table 2:** Hydrogen-bond geometry (Å, °)

*D*—H⋯*A*	*D*—H	H⋯*A*	*D*⋯*A*	*D*—H⋯*A*
O8—H8*A*⋯O13^i^	0.84	2.03	2.861 (2)	173
O8—H8*B*⋯O4^ii^	0.85	1.99	2.8130 (19)	162
O9—H9*A*⋯O7^iii^	0.85	2.16	2.9854 (17)	163
O9—H9*B*⋯O2	0.84	1.82	2.6168 (17)	159
O10—H10*A*⋯O7^ii^	0.83	2.04	2.8553 (19)	167
O10—H10*B*⋯O3^iv^	0.85	1.83	2.6615 (19)	165
O11—H11*A*⋯O7^iii^	0.89	1.90	2.7659 (18)	167
O11—H11*B*⋯O3^iv^	0.87	1.92	2.783 (2)	175
O12—H12*A*⋯O1^v^	0.84	2.11	2.9470 (18)	173
O12—H12*B*⋯O4^vi^	0.89	2.04	2.8994 (19)	163
O13—H13*A*⋯O4^ii^	0.84	1.94	2.733 (2)	157
O13—H13*B*⋯O6^vii^	0.88	2.21	2.9486 (19)	141
C7—H7⋯O11^viii^	0.93	2.50	3.371 (2)	157

Symmetry codes: (i) 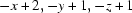; (ii) 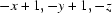; (iii) 
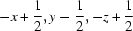
; (iv) 
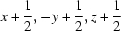
; (v) 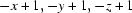; (vi) 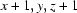; (vii) 
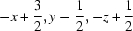
; (viii) 
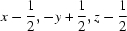
.

**Fig. 1.**
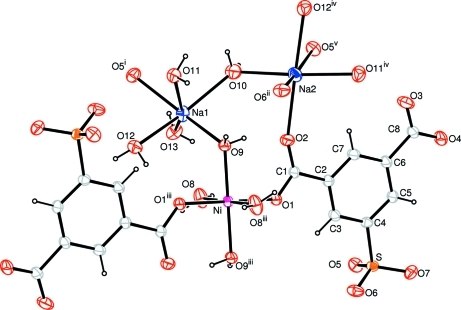
A part of the polymeric structure of the title compound with 50% probability displacement (arbitrary spheres for H atoms) [symmetry codes: (i) -x + 3/2,+y - 1/2,-z + 1/2; (ii) -x + 1/2,+y - 1/2,-z + 1/2; (iii) -x + 1,-y + 1,-z + 1; (iv) x - 1/2,-y + 1/2,+z - 1/2; (v) -x + 1,-y + 1,-z].

## supplementary crystallographic information

### Comment

As a part of investigation on π-π stacking between aromatic rings (Su & Xu, 2004; Pan *et al.*, 2006), the title Ni^II^ compound has recently been prepared in our laboratory, and its crystal structure is reported here.

A part of the three dimensional polymeric structure of the title compound is shown in Fig. 1. The Ni^II^ compound is isomorphous with the Co^II^ compound (Zhang *et al.*, 2009). The Ni atom occupies a special position in an inversion centre and assumes a distorted NiO_6_ octahedral geometry, The Ni—O bond distances (Table 1) are about 0.03 Å shorter than corresponding Co—O bond distances found in the isomorphous Co^II^ compound. Both crystallograohically indenpendent Na^I^ atoms are in distorted octahedral coordination geometry. The sulfoisophthalate trianions and water molecules bridge the metal atoms to form the polymeric structure.

The extensive O—H···O hydrogen bonding network presents in the crystal structue (Table 2), weak C—H···O hydrogen bonding also helps to stabilize the crystal structure. The distance between parallel the C2-benzne plane and C2^v^-benzene plane is 3.551 (9) Å [symmetry code: (v) 1 - *x*, 1 - *y*, -*z*], and the centroids distance between the benzene rings is 3.9349 (10) Å. These findings suggest a weak π-π stacking involving sulfoisophthlate ligand.

### Experimental

A water-ethanol solution (25 ml, 3:2) containing monosodium 5-sulfoisophthalate (0.270 g, 1 mmol), Na_2_CO_3_ (0.212 g, 2 mmol) and NiCl_2_.6H_2_O (0.600 g, 2.5 mmol) was refluxed for 8 h and filtered after cooling to room temperature. The single crystals of the title compound were obtained from the filtrate after 3 weeks.

### Refinement

Water H atoms were located in a difference Fourier map and refined as riding in as-found relative positions, with *U*_iso_(H) = 1.5*U*_eq_(O). Other H atoms were placed in calculated positions with C—H = 0.93 Å and refined in riding mode with *U*_iso_(H) = 1.2*U*_eq_(C).

### Crystal data

**Table:**

[Na_4_Ni(C_8_H_3_O_7_S)_2_(H_2_O)_12_]	*F*(000) = 876
*M**_r_* = 853.19	*D*_x_ = 1.780 Mg m^−^^3^
Monoclinic, *P*2_1_/*n*	Mo *K*α radiation, λ = 0.71073 Å
Hall symbol: -P 2yn	Cell parameters from 2356 reflections
*a* = 7.8770 (9) Å	θ = 2.5–25.0°
*b* = 17.229 (2) Å	µ = 0.90 mm^−^^1^
*c* = 11.7474 (13) Å	*T* = 295 K
β = 93.292 (4)°	Prism, green
*V* = 1591.7 (3) Å^3^	0.30 × 0.22 × 0.20 mm
*Z* = 2	

### Data collection

**Table:**

Rigaku R-AXIS RAPID IP diffractometer	3107 independent reflections
Radiation source: fine-focus sealed tube	2866 reflections with *I* > 2σ(*I*)
graphite	*R*_int_ = 0.023
Detector resolution: 10.0 pixels mm^-1^	θ_max_ = 26.0°, θ_min_ = 2.1°
ω scans	*h* = −9→9
Absorption correction: multi-scan (*ABSCOR*; Higashi, 1995)	*k* = −21→21
*T*_min_ = 0.770, *T*_max_ = 0.835	*l* = −14→14
17574 measured reflections	

### Refinement

**Table:**

Refinement on *F*^2^	Primary atom site location: structure-invariant direct methods
Least-squares matrix: full	Secondary atom site location: difference Fourier map
*R*[*F*^2^ > 2σ(*F*^2^)] = 0.025	Hydrogen site location: inferred from neighbouring sites
*wR*(*F*^2^) = 0.071	H-atom parameters constrained
*S* = 1.08	*w* = 1/[σ^2^(*F*_o_^2^) + (0.0384*P*)^2^ + 0.7337*P*] where *P* = (*F*_o_^2^ + 2*F*_c_^2^)/3
3107 reflections	(Δ/σ)_max_ = 0.001
223 parameters	Δρ_max_ = 0.42 e Å^−^^3^
0 restraints	Δρ_min_ = −0.40 e Å^−^^3^

### Special details

**Table:**

Geometry. All e.s.d.'s (except the e.s.d. in the dihedral angle between two l.s. planes) are estimated using the full covariance matrix. The cell e.s.d.'s are taken into account individually in the estimation of e.s.d.'s in distances, angles and torsion angles; correlations between e.s.d.'s in cell parameters are only used when they are defined by crystal symmetry. An approximate (isotropic) treatment of cell e.s.d.'s is used for estimating e.s.d.'s involving l.s. planes.
Refinement. Refinement of *F*^2^ against ALL reflections. The weighted *R*-factor *wR* and goodness of fit *S* are based on *F*^2^, conventional *R*-factors *R* are based on *F*, with *F* set to zero for negative *F*^2^. The threshold expression of *F*^2^ > σ(*F*^2^) is used only for calculating *R*-factors(gt) *etc*. and is not relevant to the choice of reflections for refinement. *R*-factors based on *F*^2^ are statistically about twice as large as those based on *F*, and *R*- factors based on ALL data will be even larger.

### Fractional atomic coordinates and isotropic or equivalent isotropic displacement parameters (Å^2^)

**Table:**

	*x*	*y*	*z*	*U*_iso_*/*U*_eq_	
Ni	0.5000	0.5000	0.5000	0.01887 (10)	
Na1	0.82068 (9)	0.33240 (4)	0.45637 (6)	0.02989 (18)	
Na2	0.40578 (9)	0.26823 (4)	0.20100 (6)	0.02845 (17)	
S	0.28801 (5)	0.73027 (2)	0.01772 (3)	0.01908 (11)	
O1	0.42320 (16)	0.52266 (7)	0.33588 (10)	0.0244 (3)	
O2	0.42591 (19)	0.40594 (7)	0.25407 (10)	0.0335 (3)	
O3	0.1627 (2)	0.37896 (8)	−0.12900 (12)	0.0456 (4)	
O4	0.11757 (18)	0.47912 (8)	−0.24403 (11)	0.0332 (3)	
O5	0.45777 (16)	0.74769 (7)	−0.01724 (11)	0.0279 (3)	
O6	0.25488 (18)	0.76128 (7)	0.12908 (10)	0.0291 (3)	
O7	0.15727 (16)	0.75328 (7)	−0.06942 (11)	0.0267 (3)	
O8	0.75086 (17)	0.51710 (9)	0.46157 (11)	0.0362 (3)	
H8A	0.8191	0.5403	0.5074	0.054*	
H8B	0.7789	0.5274	0.3941	0.054*	
O9	0.53288 (16)	0.38303 (6)	0.46643 (10)	0.0238 (3)	
H9A	0.4645	0.3534	0.4997	0.036*	
H9B	0.5021	0.3785	0.3975	0.036*	
O10	0.70115 (18)	0.26289 (7)	0.28653 (11)	0.0336 (3)	
H10A	0.7540	0.2629	0.2277	0.050*	
H10B	0.6997	0.2152	0.3041	0.050*	
O11	0.68483 (16)	0.21970 (8)	0.55884 (12)	0.0327 (3)	
H11A	0.5764	0.2266	0.5724	0.049*	
H11B	0.6788	0.1866	0.5033	0.049*	
O12	0.87077 (17)	0.37265 (7)	0.65035 (11)	0.0312 (3)	
H12A	0.7926	0.4047	0.6585	0.047*	
H12B	0.9552	0.3964	0.6897	0.047*	
O13	1.03972 (18)	0.40442 (8)	0.36780 (12)	0.0354 (3)	
H13A	1.0040	0.4338	0.3148	0.053*	
H13B	1.1122	0.3726	0.3378	0.053*	
C1	0.4055 (2)	0.47741 (9)	0.25112 (14)	0.0205 (3)	
C2	0.3474 (2)	0.51522 (9)	0.13980 (14)	0.0211 (3)	
C3	0.3448 (2)	0.59532 (9)	0.12908 (13)	0.0210 (3)	
H3	0.3862	0.6266	0.1889	0.025*	
C4	0.2800 (2)	0.62800 (9)	0.02841 (14)	0.0187 (3)	
C5	0.2204 (2)	0.58282 (9)	−0.06300 (14)	0.0218 (3)	
H5	0.1774	0.6060	−0.1301	0.026*	
C6	0.2258 (2)	0.50242 (9)	−0.05294 (14)	0.0224 (4)	
C7	0.2909 (2)	0.46959 (10)	0.04849 (14)	0.0244 (4)	
H7	0.2966	0.4159	0.0551	0.029*	
C8	0.1644 (2)	0.44968 (10)	−0.15014 (14)	0.0240 (4)	

### Atomic displacement parameters (Å^2^)

**Table:**

	*U*^11^	*U*^22^	*U*^33^	*U*^12^	*U*^13^	*U*^23^
Ni	0.02441 (18)	0.01777 (16)	0.01396 (16)	0.00201 (11)	−0.00293 (12)	−0.00019 (10)
Na1	0.0270 (4)	0.0368 (4)	0.0256 (4)	0.0015 (3)	−0.0011 (3)	0.0022 (3)
Na2	0.0305 (4)	0.0314 (4)	0.0234 (4)	−0.0003 (3)	0.0012 (3)	−0.0038 (3)
S	0.0229 (2)	0.01610 (19)	0.0184 (2)	−0.00051 (15)	0.00256 (15)	0.00059 (14)
O1	0.0382 (7)	0.0198 (6)	0.0145 (6)	0.0029 (5)	−0.0053 (5)	−0.0005 (5)
O2	0.0610 (9)	0.0186 (6)	0.0196 (6)	0.0057 (6)	−0.0087 (6)	0.0003 (5)
O3	0.0870 (12)	0.0189 (7)	0.0285 (7)	−0.0022 (7)	−0.0168 (7)	−0.0035 (5)
O4	0.0503 (8)	0.0298 (7)	0.0181 (6)	0.0013 (6)	−0.0090 (6)	−0.0012 (5)
O5	0.0249 (7)	0.0297 (7)	0.0294 (7)	−0.0062 (5)	0.0045 (5)	−0.0013 (5)
O6	0.0448 (8)	0.0212 (6)	0.0221 (7)	−0.0007 (5)	0.0094 (6)	−0.0036 (5)
O7	0.0272 (7)	0.0243 (6)	0.0284 (7)	0.0031 (5)	−0.0006 (5)	0.0058 (5)
O8	0.0294 (7)	0.0570 (9)	0.0223 (7)	−0.0067 (6)	0.0020 (5)	−0.0017 (6)
O9	0.0336 (7)	0.0187 (6)	0.0185 (6)	0.0011 (5)	−0.0041 (5)	−0.0004 (5)
O10	0.0472 (8)	0.0290 (7)	0.0246 (7)	0.0009 (6)	0.0034 (6)	0.0035 (5)
O11	0.0276 (7)	0.0317 (7)	0.0381 (8)	0.0043 (5)	−0.0047 (6)	−0.0062 (6)
O12	0.0337 (7)	0.0291 (7)	0.0304 (7)	0.0028 (5)	−0.0013 (6)	−0.0073 (5)
O13	0.0381 (8)	0.0312 (7)	0.0370 (8)	0.0066 (6)	0.0037 (6)	0.0064 (6)
C1	0.0265 (9)	0.0194 (8)	0.0153 (8)	0.0006 (6)	−0.0020 (6)	0.0009 (6)
C2	0.0269 (9)	0.0205 (8)	0.0155 (8)	0.0012 (7)	−0.0012 (6)	0.0005 (6)
C3	0.0268 (9)	0.0203 (8)	0.0157 (8)	−0.0007 (6)	0.0002 (6)	−0.0017 (6)
C4	0.0213 (8)	0.0166 (7)	0.0185 (8)	−0.0010 (6)	0.0024 (6)	0.0004 (6)
C5	0.0276 (9)	0.0218 (8)	0.0156 (8)	0.0006 (7)	−0.0023 (6)	0.0023 (6)
C6	0.0290 (9)	0.0219 (8)	0.0161 (8)	−0.0005 (7)	−0.0021 (7)	−0.0012 (6)
C7	0.0360 (10)	0.0170 (8)	0.0198 (8)	−0.0003 (7)	−0.0024 (7)	−0.0005 (6)
C8	0.0312 (9)	0.0221 (8)	0.0182 (8)	−0.0002 (7)	−0.0027 (7)	−0.0026 (6)

### Geometric parameters (Å, °)

**Table:**

Ni—O1^i^	2.0252 (11)	O5—Na1^vii^	2.3545 (14)
Ni—O1	2.0252 (11)	O5—Na2^vi^	2.4814 (14)
Ni—O8^i^	2.0731 (14)	O6—Na2^viii^	2.4271 (14)
Ni—O8	2.0731 (14)	O8—H8A	0.8407
Ni—O9	2.0727 (11)	O8—H8B	0.8534
Ni—O9^i^	2.0727 (11)	O9—H9A	0.8532
Na1—O5^ii^	2.3545 (14)	O9—H9B	0.8358
Na1—O12	2.3930 (14)	O10—H10A	0.8273
Na1—O13	2.4095 (16)	O10—H10B	0.8481
Na1—O9	2.4382 (14)	O11—Na2^iii^	2.3511 (15)
Na1—O10	2.4669 (15)	O11—H11A	0.8860
Na1—O11	2.5519 (16)	O11—H11B	0.8656
Na1—Na2^iii^	3.3901 (10)	O12—Na2^iii^	2.5105 (15)
Na2—O11^iv^	2.3511 (15)	O12—H12A	0.8368
Na2—O6^v^	2.4271 (14)	O12—H12B	0.8876
Na2—O2	2.4561 (14)	O13—H13A	0.8386
Na2—O5^vi^	2.4814 (14)	O13—H13B	0.8789
Na2—O10	2.4825 (16)	C1—C2	1.508 (2)
Na2—O12^iv^	2.5105 (15)	C2—C7	1.383 (2)
Na2—Na1^iv^	3.3901 (10)	C2—C3	1.386 (2)
S—O6	1.4505 (12)	C3—C4	1.381 (2)
S—O5	1.4525 (13)	C3—H3	0.9300
S—O7	1.4645 (13)	C4—C5	1.386 (2)
S—C4	1.7680 (16)	C5—C6	1.391 (2)
O1—C1	1.266 (2)	C5—H5	0.9300
O2—C1	1.242 (2)	C6—C7	1.390 (2)
O3—C8	1.244 (2)	C6—C8	1.517 (2)
O4—C8	1.250 (2)	C7—H7	0.9300
			
O1^i^—Ni—O1	180.0	O7—S—C4	107.03 (7)
O1^i^—Ni—O9	87.70 (5)	C1—O1—Ni	130.04 (11)
O1—Ni—O9	92.30 (5)	C1—O2—Na2	160.79 (11)
O1^i^—Ni—O9^i^	92.30 (5)	S—O5—Na1^vii^	136.10 (8)
O1—Ni—O9^i^	87.70 (5)	S—O5—Na2^vi^	132.45 (8)
O9—Ni—O9^i^	180.000 (1)	Na1^vii^—O5—Na2^vi^	88.98 (5)
O1^i^—Ni—O8^i^	90.09 (5)	S—O6—Na2^viii^	152.77 (8)
O1—Ni—O8^i^	89.91 (5)	Ni—O8—H8A	120.7
O9—Ni—O8^i^	91.97 (5)	Ni—O8—H8B	122.2
O9^i^—Ni—O8^i^	88.03 (5)	H8A—O8—H8B	107.8
O1^i^—Ni—O8	89.91 (5)	Ni—O9—Na1	118.93 (6)
O1—Ni—O8	90.09 (5)	Ni—O9—H9A	114.0
O9—Ni—O8	88.03 (5)	Na1—O9—H9A	114.9
O9^i^—Ni—O8	91.97 (5)	Ni—O9—H9B	104.1
O8^i^—Ni—O8	180.0	Na1—O9—H9B	98.0
O5^ii^—Na1—O12	79.14 (5)	H9A—O9—H9B	103.4
O5^ii^—Na1—O13	85.05 (5)	Na1—O10—Na2	127.93 (6)
O12—Na1—O13	100.31 (5)	Na1—O10—H10A	119.3
O5^ii^—Na1—O9	151.89 (5)	Na2—O10—H10A	99.6
O12—Na1—O9	87.31 (5)	Na1—O10—H10B	106.5
O13—Na1—O9	121.84 (5)	Na2—O10—H10B	96.3
O5^ii^—Na1—O10	100.75 (5)	H10A—O10—H10B	102.5
O12—Na1—O10	160.96 (6)	Na2^iii^—O11—Na1	87.39 (5)
O13—Na1—O10	98.64 (5)	Na2^iii^—O11—H11A	122.8
O9—Na1—O10	84.27 (5)	Na1—O11—H11A	114.7
O5^ii^—Na1—O11	73.06 (5)	Na2^iii^—O11—H11B	126.9
O12—Na1—O11	79.75 (5)	Na1—O11—H11B	98.8
O13—Na1—O11	157.76 (5)	H11A—O11—H11B	102.2
O9—Na1—O11	80.39 (5)	Na1—O12—Na2^iii^	87.44 (5)
O10—Na1—O11	82.02 (5)	Na1—O12—H12A	102.6
O5^ii^—Na1—Na2^iii^	47.04 (4)	Na2^iii^—O12—H12A	133.0
O12—Na1—Na2^iii^	47.71 (4)	Na1—O12—H12B	135.0
O13—Na1—Na2^iii^	121.18 (4)	Na2^iii^—O12—H12B	104.7
O9—Na1—Na2^iii^	106.32 (4)	H12A—O12—H12B	99.8
O10—Na1—Na2^iii^	119.01 (4)	Na1—O13—H13A	114.4
O11—Na1—Na2^iii^	43.85 (3)	Na1—O13—H13B	110.5
O11^iv^—Na2—O6^v^	100.89 (5)	H13A—O13—H13B	106.1
O11^iv^—Na2—O2	97.43 (5)	O2—C1—O1	125.48 (15)
O6^v^—Na2—O2	82.41 (5)	O2—C1—C2	118.99 (14)
O11^iv^—Na2—O5^vi^	74.44 (5)	O1—C1—C2	115.47 (14)
O6^v^—Na2—O5^vi^	169.25 (5)	C7—C2—C3	119.56 (15)
O2—Na2—O5^vi^	107.62 (5)	C7—C2—C1	119.68 (15)
O11^iv^—Na2—O10	158.30 (6)	C3—C2—C1	120.73 (14)
O6^v^—Na2—O10	100.73 (5)	C4—C3—C2	119.12 (15)
O2—Na2—O10	83.55 (5)	C4—C3—H3	120.4
O5^vi^—Na2—O10	84.57 (5)	C2—C3—H3	120.4
O11^iv^—Na2—O12^iv^	81.40 (5)	C3—C4—C5	121.77 (15)
O6^v^—Na2—O12^iv^	95.27 (5)	C3—C4—S	117.02 (12)
O2—Na2—O12^iv^	177.18 (6)	C5—C4—S	121.10 (12)
O5^vi^—Na2—O12^iv^	74.58 (5)	C4—C5—C6	119.09 (15)
O10—Na2—O12^iv^	98.50 (5)	C4—C5—H5	120.5
O11^iv^—Na2—Na1^iv^	48.76 (4)	C6—C5—H5	120.5
O6^v^—Na2—Na1^iv^	125.80 (4)	C7—C6—C5	119.09 (15)
O2—Na2—Na1^iv^	135.72 (4)	C7—C6—C8	119.18 (14)
O5^vi^—Na2—Na1^iv^	43.98 (3)	C5—C6—C8	121.73 (15)
O10—Na2—Na1^iv^	117.34 (4)	C2—C7—C6	121.34 (15)
O12^iv^—Na2—Na1^iv^	44.84 (3)	C2—C7—H7	119.3
O6—S—O5	113.27 (8)	C6—C7—H7	119.3
O6—S—O7	112.03 (8)	O3—C8—O4	124.58 (16)
O5—S—O7	111.61 (8)	O3—C8—C6	116.32 (15)
O6—S—C4	107.10 (7)	O4—C8—C6	119.10 (15)
O5—S—C4	105.26 (8)		

Symmetry codes: (i) −*x*+1, −*y*+1, −*z*+1; (ii) −*x*+3/2, *y*−1/2, −*z*+1/2; (iii) *x*+1/2, −*y*+1/2, *z*+1/2; (iv) *x*−1/2, −*y*+1/2, *z*−1/2; (v) −*x*+1/2, *y*−1/2, −*z*+1/2; (vi) −*x*+1, −*y*+1, −*z*; (vii) −*x*+3/2, *y*+1/2, −*z*+1/2; (viii) −*x*+1/2, *y*+1/2, −*z*+1/2.

### Hydrogen-bond geometry (Å, °)

**Table:**

*D*—H···*A*	*D*—H	H···*A*	*D*···*A*	*D*—H···*A*
O8—H8A···O13^ix^	0.84	2.03	2.861 (2)	173
O8—H8B···O4^vi^	0.85	1.99	2.8130 (19)	162
O9—H9A···O7^v^	0.85	2.16	2.9854 (17)	163
O9—H9B···O2	0.84	1.82	2.6168 (17)	159
O10—H10A···O7^vi^	0.83	2.04	2.8553 (19)	167
O10—H10B···O3^iii^	0.85	1.83	2.6615 (19)	165
O11—H11A···O7^v^	0.89	1.90	2.7659 (18)	167
O11—H11B···O3^iii^	0.87	1.92	2.783 (2)	175
O12—H12A···O1^i^	0.84	2.11	2.9470 (18)	173
O12—H12B···O4^x^	0.89	2.04	2.8994 (19)	163
O13—H13A···O4^vi^	0.84	1.94	2.733 (2)	157
O13—H13B···O6^ii^	0.88	2.21	2.9486 (19)	141
C7—H7···O11^iv^	0.93	2.50	3.371 (2)	157

Symmetry codes: (ix) −*x*+2, −*y*+1, −*z*+1; (vi) −*x*+1, −*y*+1, −*z*; (v) −*x*+1/2, *y*−1/2, −*z*+1/2; (iii) *x*+1/2, −*y*+1/2, *z*+1/2; (i) −*x*+1, −*y*+1, −*z*+1; (x) *x*+1, *y*, *z*+1; (ii) −*x*+3/2, *y*−1/2, −*z*+1/2; (iv) *x*−1/2, −*y*+1/2, *z*−1/2.
